# Kv1.3 channel blocker (ImKTx88) maintains blood–brain barrier in experimental autoimmune encephalomyelitis

**DOI:** 10.1186/s13578-017-0158-2

**Published:** 2017-06-07

**Authors:** Jie Huang, Song Han, Qi Sun, Yipeng Zhao, Junchen Liu, Xiaolu Yuan, Wenqian Mao, Biwen Peng, Wanhong Liu, Jun Yin, Xiaohua He

**Affiliations:** 1 Department of Pathophysiology, School of Basic Medical Sciences, Wuhan University, Wuhan, No. 185, Donghu Road, Wuchang District, Wuhan, 430071 China; 2 Hubei Provincial Key Laboratory of Developmentally Originated Disease, School of Basic Medical Sciences, Wuhan University, No. 185, Donghu Road, Wuchang District, Wuhan, 430071 China; 3 Hubei Province Key Laboratory of Allergy and Immunology, School of Basic Medical Sciences, Wuhan University, No. 185, Donghu Road, Wuchang District, Wuhan, 430071 China

**Keywords:** Kv1.3 channel blocker, Blood–brain barrier, Experimental autoimmune encephalomyelitis, Multiple sclerosis, IL-17

## Abstract

**Background:**

Disruption of blood–brain barrier (BBB) and subsequent infiltration of auto-reactive T lymphocytes are major characteristics of multiple sclerosis (MS) and experimental autoimmune encephalomyelitis (EAE). Kv1.3 channel blockers are demonstrated potential therapeutic effects on MS patients and EAE models, maybe via reducing activation of T cells. However, it remains to be explored whether Kv1.3 channel blockers maintain integrity of BBB in MS model.

**Results:**

In this study, ImKTx88, a highly selective Kv1.3 channel blocker, was used to determine the role of Kv1.3 channel in the pathogenesis of EAE, particularly in the maintenance of BBB. ImKTx88 ameliorated pathological severity in the EAE rats, and reduced extravasation into CNS. ImKTx88 also ameliorated the severity of loss or redistribution of tight junction proteins, and inhibited over-expression of ICAM-1 and VCAM-1 in the brain from EAE rats. Furthermore ImKTx88 protection was associated with activation of Ang-1/Tie-2 axis, and might be due to decreased IL-17 production.

**Conclusions:**

ImKTx88 may be a novel therapeutic agent for MS treatment by stabilizing the BBB.

**Electronic supplementary material:**

The online version of this article (doi:10.1186/s13578-017-0158-2) contains supplementary material, which is available to authorized users.

## Background

Multiple sclerosis (MS) is a typical neuroinflammatory demyelinating disease characterized by breakdown of blood–brain barrier (BBB) and infiltration of activated myelin-reactive T cells into the parenchyma of the central nervous system (CNS) [1]. Experimental autoimmune encephalomyelitis (EAE) is the most commonly used model in MS research [2]. BBB disruption is a unique feature during the development of MS [3], usually accompanied with demyelination and proinflammatory cytokine production in both MS patients and EAE animals [4, 5].

Blood–brain barrier is a brain-specific capillary barrier that is primarily formed by microvascular endothelial cells, which are surrounded by basement membranes, pericytes and astrocytes [6]. Tight junctions in endothelial cells are important in the formation of the impermeable barrier [7]. Tight junctions are composed of a complex of transmembrane tight junction (TJ) proteins, including claudin-5, occludin and zonula occludens protein 1 (ZO-1) [8]. Loss of TJ proteins may occur when BBB is disrupted [9]. Permeability of BBB is increased at the early stage of MS [10, 11], correlating with loss of tight junctions and up-regulation of endothelial adhesion molecules [intercellular adhesion molecule 1 (ICAM-1) and vascular cell adhesion molecule 1 (VCAM-1)] [12]. Angiopoietin-1 (Ang-1), one of angiopoietins derived from perivascular cells, binds to the endothelial-specific receptor tyrosine kinase 2 (Tie-2) for maintaining integrity of BBB [13]. In the early stage of EAE, the expression of Ang-1 and Tie-2 is suppressed in the local inflammatory lesions [14]. Increased interleukin-17 (IL-17) production from activated Th17 cells contributes to the pathogenesis of MS and EAE [15], consequently disrupting BBB tight junctions in vivo and in vitro [16].

Kv1.3 channel, a voltage-gated K^+^ channel, is opened in response to membrane depolarization for the surrounding increased extracellular K^+^ [17]. Kv1.3 channel is highly expressed in IL-17^+^CCR7^−^ Th17 cells [18]. It is reported that the density of Kv1.3 channel is increased in myelin-reactive T cells from MS patients [19]. Specific Kv1.3 channel blockers relieve autoimmune and metabolic symptoms in animal disease models, such as autoimmune diseases, chronic inflammatory diseases and metabolic diseases, without obvious side effects [20]. Moreover, specific Kv1.3 channel blockers exhibit effective results in preclinical trials [20], showing improve the field of vision and motor skills for the majority of MS patients [21]. Therefore, selective Kv1.3 channel blockers may be potential therapeutic drugs for MS and EAE [22]. To investigate the mechanisms underlying the therapeutic effects of Kv1.3 channel blockers, most studies focus on inhibition of auto-reactive effector memory T cell activation by suppressing influx of Ca^2+^ for controlling T cell proliferation [23]. However little is known about the modulation effects of Kv1.3 channel blockers on BBB. In our previous study, ImKTx88, a highly selective Kv1.3 channel blocker derived from the scorpion *Isometrus maculates*, exhibits good selectivity for Kv1.3 channel over Kv1.1 channel [24]. In this study, the protection role of ImKTx88 in maintaining BBB in EAE was investigated. Our data may provide useful information for both basic research in EAE and clinical intervention in MS.

## Results

### Kv1.3 channel blocker (ImKTx88) ameliorates pathological changes in EAE

**Fig. 1:**
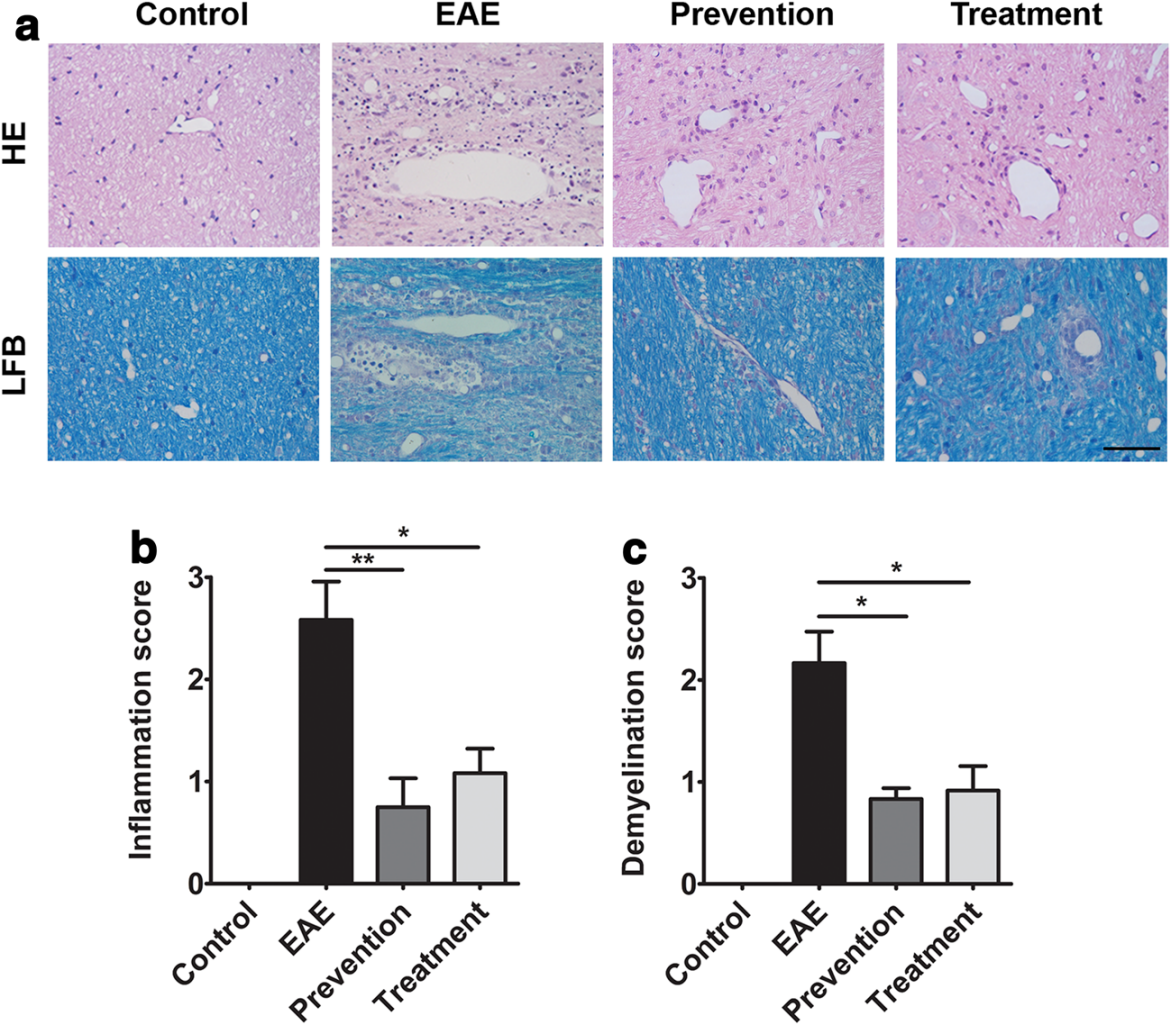
ImKTx88 ameliorates inflammation and demyelination in EAE rats. **a** HE and LFB staining of cerebellum sections demonstrated inflammation and demyelination on day 21 after EAE induction. The *scale bar* represents 100 µm; **b** the inflammation score and **c** demyelination score were evaluated. Data represent the mean ± SEM, *P < 0.05, **P < 0.01

Experimental autoimmune encephalomyelitis model was used to evaluate whether ImKTx88 is a potential therapeutic drug for MS patients. Because cerebellum is a main inflamed area in the EAE rat brain [25], histopathology of cerebellum were employed to assess the severity of EAE using hematoxylin–eosin (HE) and Luxol fast blue (LFB) staining. Compared to control, it was detected that extensive perivascular cuffing with inflammatory cells around the white matter of cerebellum from the EAE rats (Fig. 1a, b). Interestingly cellular infiltration was significantly decreased in ImKTx88 prevention (0.75 ± 0.28 vs. 2.58 ± 0.37, *P* < 0.01) or ImKTx88 treated (1.08 ± 0.24 vs. 2.58 ± 0.37, *P* < 0.05) EAE rats. In addition, no significant difference was observed between the ImKTx88 prevention and treatment groups (Fig. 1b). In the EAE group, a large amount of demyelination was observed in the cerebellum, especially in white matter (Fig. 1a, c). Demyelination score was significantly lower in the ImKTx88 prevention group (0.83 ± 0.26 vs. 2.17 ± 0.75, *P* < 0.05) or the ImKTx88 treated group (0.92 ± 0.58 vs. 2.17 ± 0.75, *P* < 0.05), compared to that from the EAE group. No significant different demyelination was observed between the prevention and the treatment groups. Consistent with histopathology, both the prevention and the treatment groups exhibited lower clinical scores than the EAE group (Additional file 1: Table S1).

### ImKTx88 maintains BBB integrity following EAE induction

**Fig. 2:**
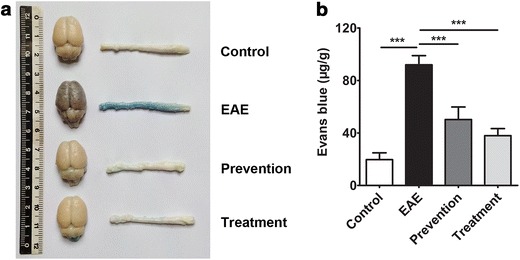
ImKTx88 protects the BBB in EAE rats. **a** Macroscopic images of whole brains and spinal cords (n = 6) on day 21 after EAE induction. Evans blue was injected into the tail vein 60 min before euthanization, and the brains, as well as the lumbar enlargements, were removed to detect Evans blue extravasation in the four groups. **b** Quantification of Evans blue dye extracted from homogenized samples. Data are representative of three independent experiments and represent the mean ± SEM, ***P < 0.001

Destruction of BBB is a key initiating event in MS, Evans blue extravasation was used to detect BBB permeability. Evans blue was retained in the systemic circulation of the control group, confirming vascular integrity; whereas in the EAE rats, a large amount of Evans blue was diffused to cerebral cortex, cerebellum and spinal cord, indicating vascular leakage. However, BBB leakage was inhibited in ImKTx88 treated rats (Fig. 2a). Evens blue was >fourfold higher in cerebellum and spinal cord from EAE rats than that from control rats (91.93 ± 7.05 μg/g vs. 19.68 ± 5.18 μg/g, *P* < 0.001). Abnormal Evans blue extraction in EAE rats was significantly attenuated with ImKTx88 prevention (50.24 ± 9.56 μg/g vs. 91.93 ± 7.05 μg/g, *P* < 0.001) or treatment (37.96 ± 5.37 μg/g vs. 91.93 ± 7.05 μg/g, *P* < 0.001) (Fig. 2b).

### ImKTx88 ameliorates EAE-induced loss of tight junction proteins

**Fig. 3:**
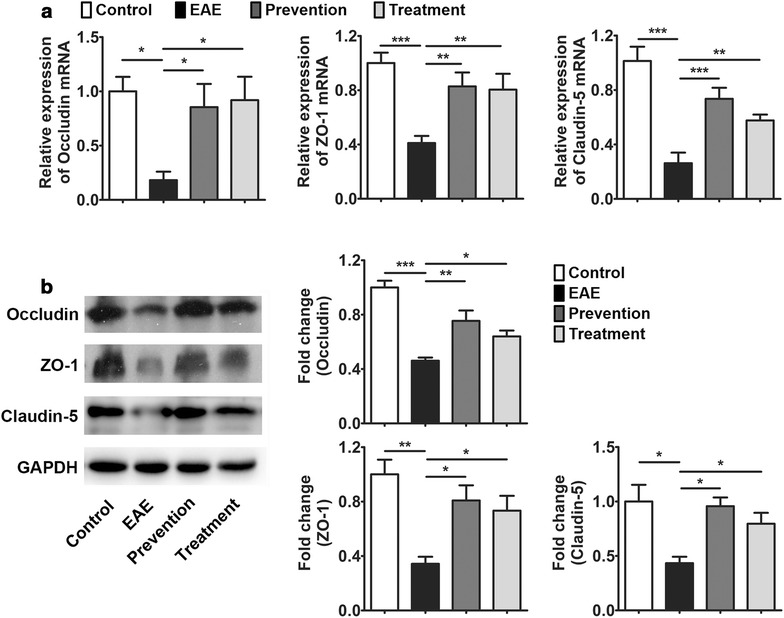
ImKTx88 ameliorates the EAE-induced loss of the TJ proteins. **a** The relative gene expression of occludin, ZO-1 and claudin-5 in cerebellar white matter were measured by qRT-PCR (n = 8–12). **b** Protein levels of occludin, ZO-1 and claudin-5 were detected via western blot (n = 4). Band densities were normalized to GAPDH and quantified. Experiments were repeated in triplicate. Data represent the mean ± SEM, *P < 0.05, **P < 0.01, ***P < 0.001

Decreased expression or abnormal localization of TJ proteins has been described in MS, and these pathological changes are tightly associated with BBB leakage [4]. To further explore the protective effects of ImKTx88 on the BBB, primary TJ proteins, occludin, ZO-1 and claudin-5 were quantified. qRT-PCR demonstrated that the occludin, ZO-1 and claudin-5 mRNA levels were decreased in cerebellum from EAE rats induction (Fig. 3a). However EAE induced suppression of occludin, ZO-1 and claudin-5 mRNA was restored in both ImKTx88 prevention and treatment groups (Fig. 3a). Consistent with mRNA expression, the protein levels of occludin, ZO-1 and claudin-5 in EAE group were also suppressed, but were restored with ImKTx88 intervention (Fig. 3b).

### ImKTx88 treatment restores the EAE-induced irregular morphology of claudin-5

**Fig. 4:**
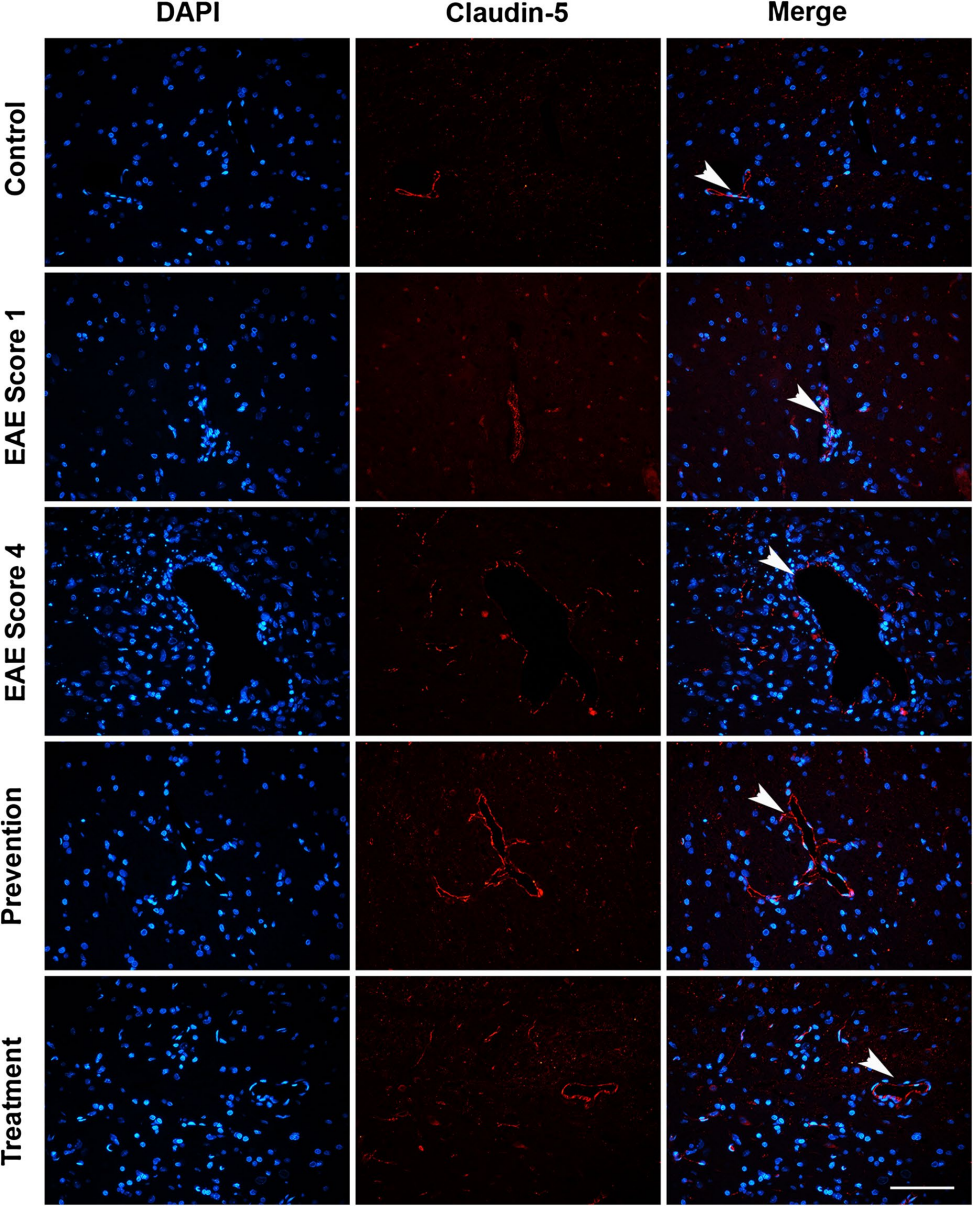
The effects of ImKTx88 on the morphological change of claudin-5 during EAE. Immunofluorescence was performed with a claudin-5 antibody (*red*) and DAPI staining (*blue*). The *scale bar* represents 100 μm. Claudin-5 in the control group showed a linear-like appearance and continuous junctional pattern (*arrowhead*). In EAE score 1 rats, the junctional pattern became discontinuous, and microvessels were surrounded with *rows* of claudin-5-positive fluorescent aggregates (*arrowhead*). In EAE score 4 rats, the linear pattern of claudin-5 was lost, and only some short claudin-5 positive tracts (*arrowhead*) could be detected. ImKTx88 prevention and treatment up-regulated claudin-5 expression and maintained the continuous linear-like appearance (*arrowheads*)

In MS patients and EAE animal models, the distribution of tight junctions is altered [4]. In our study, claudin-5 in the control group showed a linear-like appearance and continuous junctional pattern observed in microvessels of the cerebellum white matter (Fig. 4). In cerebellar demyelination lesions of mild EAE rats (EAE score 1), the junctional pattern became discontinuous, and microvessels were surrounded by rows of claudin-5-positive fluorescent aggregates. In the severe EAE rats (EAE score 4), the linear pattern of claudin-5 was lost, and only some short claudin-5 positive tracts was detected. Both ImKTx88 pre-treatment (prevention) and treatment after EAE induction (treatment) restored the lost claudin-5 and the rearrangement of the irregular morphology of claudin-5.

### ImKTx88 decreases EAE-induced ICAM-1 and VCAM-1

**Fig. 5:**
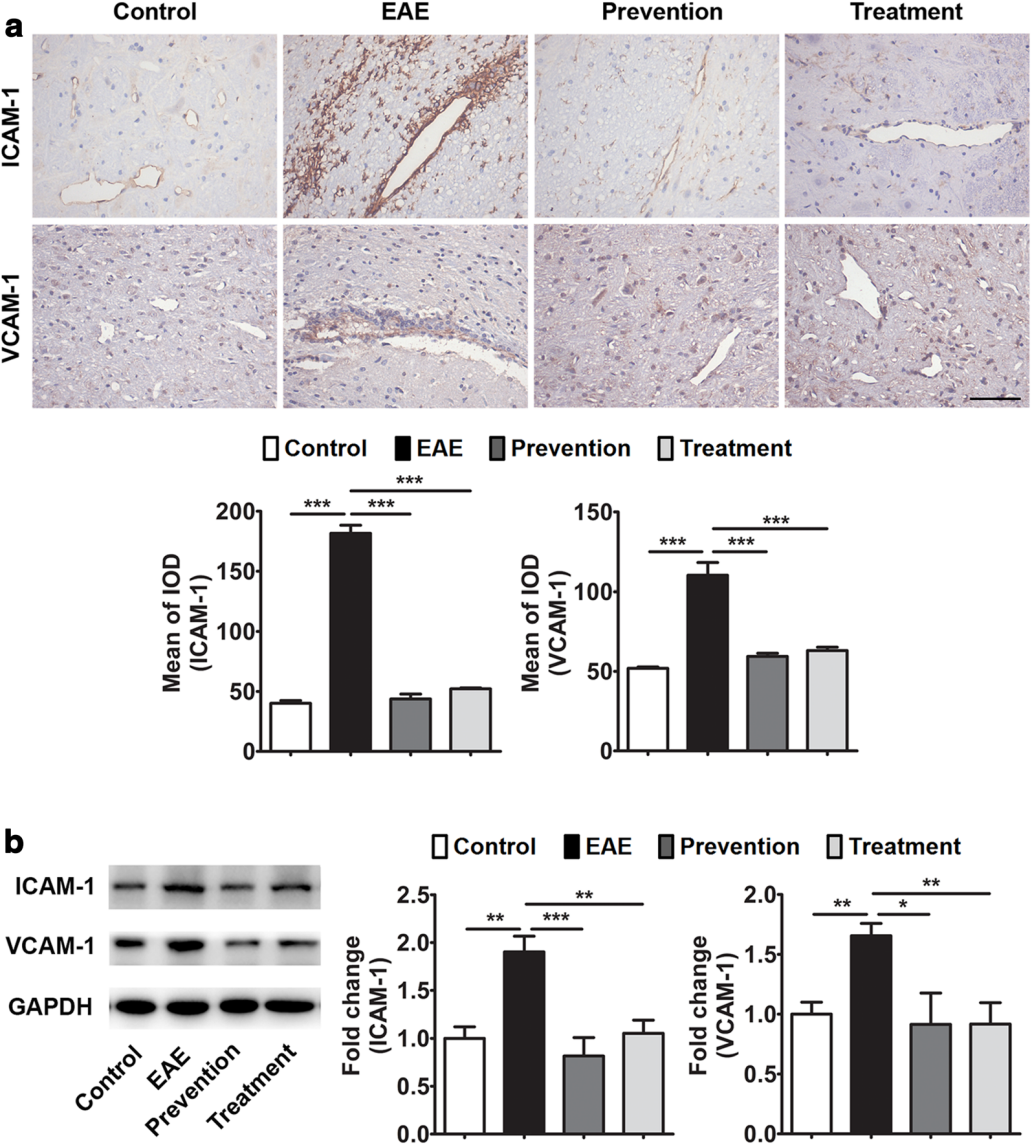
Effects of ImKTx88 on ICAM-1 and VCAM-1 expression. **a** Representative immunohistochemistry of ICAM-1 and VCAM-1 expression in the cerebellar white matter of EAE-induced and ImKTx88 treated rats. The *scale bar* represents 100 µm. Staining was quantified by mean of the integrated optical density (IOD) (5 random images per section and n = 3). **b** A representative western blot of cerebellum homogenates and quantification analysis revealed that the levels of ICAM-1 and VCAM-1 were significantly increased in EAE-induced rats, and ImKTx88 could decrease the levels of ICAM-1 and VCAM-1 (n = 5–12). GAPDH was used as an internal loading control. Data represent the mean ± SEM, *P < 0.05, **P < 0.01, ***P < 0.001

To further investigate the mechanism underlying BBB protection in ImKTx88 treated rats, we examined the expression of ICAM-1 and VCAM-1. Immunohistochemistry showed significantly higher ICAM-1 (181.6 ± 6.75 vs. 40.08 ± 2.29 IOD, *P* < 0.001) and VCAM-1 (110.2 ± 7.96 vs. 51.88 ± 0.92 IOD, *P* < 0.001) in the perivascular space of the cerebellar white matter from the EAE group than in the control group (Fig. 5a). As expected, both ImKTx88 prevention and treatment decreased ICAM-1 (prevention: 43.76 ± 4.01 vs. 181.6 ± 6.75 IOD, *P* < 0.001; treatment: 52.17 ± 0.81 vs. 181.6 ± 6.75 IOD, *P* < 0.001) and VCAM-1 (prevention: 59.44 ± 1.96 vs. 110.2 ± 7.96 IOD, *P* < 0.001; treatment: 63.09 ± 2.09 vs. 110.2 ± 7.96 IOD, *P* < 0.001) on vascular endothelium with EAE induction. Western blot further confirmed these results by measuring protein levels of ICAM-1 and VCAM-1 from whole cerebellum homogenate (Fig. 5b). The inhibitory effects on these two adhesion molecules between the ImKTx88 prevention group and the treatment group showed no significant difference.

### ImKTx88 activates Ang-1/Tie-2 axis in the cerebellum after EAE induction

**Fig. 6:**
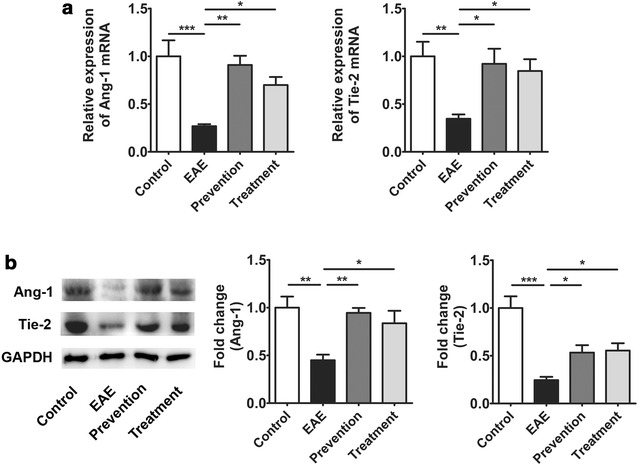
Effects of ImKTx88 treatment on the expression of Ang-1 and Tie-2. **a** Relative expression of Ang-1 and Tie-2 mRNA from the control group, EAE group, ImKTx88 prevention group and ImKTx88 treatment group (n = 6–8) were measured by qRT-PCR. **b** Western blot showed that levels of Ang-1 and Tie-2 were significantly decreased when EAE was induced, and ImKTx88 could increase their expression (n = 5). GAPDH was used as an internal control. Data are presented as the mean ± SEM, *P < 0.05, **P < 0.01, ***P < 0.001

The protective effects of Ang-1/Tie-2 axis up-regulation on the BBB have been confirmed by promoting the expression of TJ proteins and inhibiting the expression of cellular adhesion molecules after brain injury [26]. Herein, the expression of Ang-1 and Tie-2 in the cerebellum were measured after EAE induction and ImKTx88 treatment. The mRNA (Fig. 6a) and protein levels (Fig. 6b) of Ang-1 and Tie-2 were both decreased in the EAE group when compared to the control group. Both ImKTx88 prevention and treatment up-regulated Ang-1 and Tie-2 at mRNA and protein level to a certain extent.

### ImKTx88 reduces IL-17 production in vivo and in vitro

**Fig. 7:**
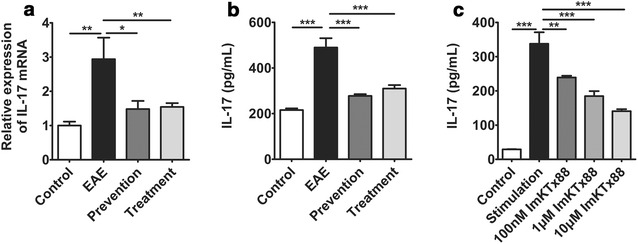
ImKTx88 treatment decreases IL-17 expression in vivo and in vitro. **a** mRNA levels of IL-17 from the control group, EAE group, ImKTx88 prevention group and ImKTx88 treatment group (n = 8) were analyzed by qRT-PCR. **b** IL-17 expression was analyzed via ELISA. EAE induction significantly increased the levels of IL-17 in the cerebellum, and ImKTx88 inhibited the increase in IL-17. **c** PBMCs were pretreated with ImKTx88 (100 nM, 1 and 10 μM) for 60 min and then stimulated with Con A (100 μg/ml) for 24 h. After treatment, IL-17 was detected in culture medium using ELISA. Compared with Con A stimulated cells, the PBMCs pretreated with ImKTx88 showed low expression of IL-17. Data represent the mean ± SEM, *P < 0.05, **P < 0.01, ***P < 0.001

We previously demonstrated that another selective Kv1.3 channel blocker has an inhibitory effect on Th17 cell activation [18]. Thus we evaluated the effect of ImKTx88 on IL-17 production in EAE rats. Both the mRNA and protein levels of IL-17 were >twofold higher in the EAE group than in the control group, and IL-17 expression was inhibited by the administration of ImKTx88 during prevention or treatment after EAE induction (Fig. 7a, b). To investigate the effect of ImKTX88 on IL-17 secretion in vitro, peripheral blood mononuclear cells (PBMCs) were treated with ImKTx88 at different concentrations before Concanavalin A (Con A) stimulation, and the levels of IL-17 in the supernatant were greatly decreased after ImKTx88 treatment (*P* < 0.01) (Fig. 7c), showing a dose-dependent manner.

## Discussion

In this study, we demonstrate that the selective Kv1.3 channel blocker ImKTx88 has a therapeutic effect on EAE via protecting BBB, which was associated with the activation of the Ang-1/Tie-2 axis and reduced activation of Kv1.3^high^ Th17 cells.

Previous studies report the potential therapeutic effects of selective Kv1.3 channel blockers on MS and EAE, via specific inhibition of effector memory T lymphocyte (T_EM_) proliferation [19, 22]. Short peptides derived or modified from scorpion venom or sea anemone extracts selectively inhibit T cell activation without obvious side effects during EAE [21]. Scorpion venom peptide ImKTx88 exhibits a higher selectivity for Kv1.3 over Kv1.1 (4200-fold) [27], which makes it stand out from other selective Kv1.3 channel blockers such as scorpion toxin HsTX1 (more than 2000-fold for Kv1.3 over Kv1.1) [28] and sea anemone toxin ShK-Q16K-PEG [20K] (1600-fold for Kv1.3 over Kv1.1) [29]. In this study, we demonstrated that ImKTx88 successfully attenuated the pathogenesis of EAE in rats, including neuroinflammation and demyelination with clinical impact. Our results confirm the therapeutic effect of Kv1.3 channel blockers on EAE. However, only a few studies have focused on BBB stabilization.

BBB plays a key role in protecting the homeostatic balance of CNS and preventing the entry of potentially neurotoxic substances, including leukocytes and pathogens, into the brain under physiological conditions [6]. Disruption of BBB in MS and EAE [4] is related to two main processes. The first is due to disruption of integrity, resulting in BBB leakage and alteration of junctional components. The second is due to BBB activation, which is mainly correlated with endothelial activation and the expression of adhesion molecules [30, 31]. Increased BBB permeability is the hallmark of acute inflammatory lesions in MS patients [12], and appears in the periventricular normal-appearing white matter of MS patients using dynamic contrast-enhanced MRI imaging [32]. A correlation between BBB permeability and perivascular inflammatory cuffing is observed at an early stage of an EAE marmoset model [33]. Those findings are consistent with our current study as such: the markedly increased extravasation of the Evans blue and subsequent inflammatory infiltration were detected following induction of EAE, confirming that BBB integrity was compromised in EAE rats. Interestingly, no significant different protective effect was observed between ImKTx88 prevention and ImKTx88 treatment groups. Our explanation is such: protective effect of ImKTx88 prevention group is due to reduced inflammation in EAE, and consequently reduced BBB disruption. Our results demonstrated that ImKTx88 reduced Evans blue extravasation and the areas of inflammatory lesions, which suggested that ImKTx88 exerted a protective effect via reducing BBB leakage. Whereas, such protective effect of ImKTx88 treatment is like to restore disrupted BBB efficiently.

The integrity of BBB is depended on an intact junction complex. TJ proteins are the major component of the junction complex, forming a seal in the endothelial cells to prevent solutes from undergoing paracellular diffusion and to mediate the gate function of the BBB [8]. Loss of TJ proteins leads to BBB breach and increases permeability of BBB. It is reported that BBB leakage is related to alteration of TJ proteins in MS patients [10]. This is consistent with our results in EAE rats, demonstrating that increased Evans blue extravasation was associated with the decreased expression of TJ proteins (occludin, ZO-1 and claudin-5) during EAE. ImKTx88 inhibited BBB permeability via up-regulating TJ proteins. Our finding is in line with others, showing a correlation between BBB integrity protection and TJ proteins in a Tanshinone IIA treated EAE model [34]. In addition to the levels of TJ proteins, the redistribution of TJ proteins may also exacerbate BBB leakage [10]. Focal reorganization of ZO-1 and claudin-5 are observed in the cerebellum of relapsing-remitting MS patients, as well as in a mouse EAE model [4]. Claudin-5, the main constituent of tight junctions, is mostly localized at the cell boundaries. Claudin-5 is redistributed during the development of local inflammation, resulting in BBB disruption [35]. These observations are supporting our current finding, showing similar morphological changes and the focal degradation of claudin-5 [36]. More recently it is found that claudin-5 participate in leukocyte infiltration [37], which may explain why claudin-5 was significantly modified in cerebellum of current EAE rats. We also found that the degree of claudin-5 disorganization was positive correlated with the clinical scores, which is similar to the modification of claudin-5 in relapsing-remitting MS patients [4]. Sulforaphane preserves BBB via improving the distribution of TJ proteins in EAE mice [38]. In our current study, ImKTx88 increased the expression of TJ proteins and maintained the linear-like structure of claudin-5 (Fig. 4), suggesting that ImKTx88 plays a similar protective role for BBB during the development of EAE.

In addition to the disturbed BBB integrity, endothelial cell activation is another key factor contributing to BBB dysfunction. High levels of ICAM-1 and VCAM-1 are considered hallmarks of activated endothelial cells in vitro and in vivo [39]. Endothelial cells express intercellular adhesion molecules, which mediate the adhesion process and transmigration of leukocytes and lymphocytes to CNS, and subsequently increased inflammation, contributing to increased BBB disruption and EAE severity [4, 40]. Furthermore, in cultured primary human brain microvascular endothelium, soluble VCAM-1 increased brain endothelial permeability and altered tight junction morphology [41]. Our results showed that ImKTx88 inhibited up-regulation of ICAM-1 and VCAM-1 in the cerebellum from EAE rats, suggesting the protective role of ImKTx88 for BBB is due to reducing endothelial cell activation. An inhibitory effect on the VCAM-1 of T cells is also observed following treatment with MgTX (another Kv1.3 blocker) [42]. The effects of decreasing adhesion molecule levels by ImKTx88 may be similar to the effects of MgTX.

Ang-1 and its receptor Tie-2 are closely related to the function and maintenance of BBB [43]. Ang-1 prevents the adult vasculature leakage [44], and the activation of the pericyte-derived Ang-1/Tie-2 pathway induces occludin expression in a BBB model [45]. In EAE mice, both Ang-1 and Tie-2 are reduced at an early stage [14]. Furthermore, Ang-1 regulates brain endothelial permeability through PTPN-2 mediated dephosphorylation of occludin in human brain microvascular endothelial cells [46]. This is line with our current study, showing down-regulated Ang-1 and Tie-2 at mRNA and protein levels, accompanied with severely broken TJ structure at the peak stage of EAE. The data suggests that down-regulation of Ang-1/Tie-2 axis contributes to BBB disruption. It is also reported that activation of Ang-1/Tie-2 axis alleviates BBB leakage and promote TJ protein expression after traumatic brain injury [26]. Such finding is consistent with our current studies showing Ang-1 and Tie-2 were increased after ImKTx88 treatment. Our results suggest that ImKTx88 enhances vascular TJ protein restoration via activating Ang-1/Tie-2 axis. Ang-1, as a member of the endothelial growth factors family, has been used for EAE treatment as anti-inflammatory agent [47] via reducing the expression of ICAM-1 and VCAM-1 on endothelial cells [48]. In consideration of the inhibitory effects of Ang-1/Tie-2 on adhesion molecules, ImKTx88 may act as a stabilization of BBB via promoting activation Ang-1/Tie-2 in our EAE rats. However, the precisely role of Kv1.3 channel blockers in BBB protection remained to be revealed.

Th17 cell secreted IL-17 is highly expressed in the CNS lesions of MS patients [49], and IL-17 contributes to the disruption of the BBB during EAE [16]. IL-17 disrupted BBB via up-regulating reactive oxygen species (ROS) production, but down-regulating occludin expression in EAE mice [50]. Sulforaphane and rifampicin protect BBB disruption and attenuate EAE via inhibiting Th17 cells differentiation [38, 51]. In addition IL-17 is a pro-inflammatory cytokine, and neuroinflammation results in a decrease in Ang-1 production [52]. Increased IL-17 accompanied with decreased Ang-1 is observed during acute kidney injury in critically ill patients [53], accompanied with microvessel disruption. Our previous study found that activation of Th17 cells is inhibited by a selective Kv1.3 channel blocker [18]. Our current study demonstrated that the selective Kv1.3 channel blocker ImKTx88 down-regulated Th17 cell activation and inhibited IL-17 production in vivo and in vitro. Therefore, we hypothesize that ImKTx88 treatment reduces BBB disruption and enhances Ang-1/Tie-2 axis activity, via inhibition of Th17 cells.

## Conclusions

In summary, we proposed that ImKTx88 blocks the Kv1.3 channel in Th17 cells and the production of IL-17, which may enhance the activity of Ang-1/Tie-2 axis. Subsequently, ImKTx88 stabilizes BBB via restoration of tight junctions and reduces activation of endothelial cells. Finally ImKTx88 ameliorates pathological changes in EAE. All of these findings suggest that ImKTx88 maybe a novel therapeutic agent in selective Kv1.3 channel blocker for the prevention and/or treatment of MS.

## Methods

### Animals

Female SD rats between 6 and 8 weeks of age were obtained from the ABSL-III laboratory at Wuhan University and were raised under specific pathogen-free conditions. All animals were treated based on protocols approved by the Institutional Animal Care and Use Committee.

### Rat EAE induction and ImKTx88 treatment

ImKTx88 was produced as described previously [43], and ImKTx88 activity was measured prior to ImKTx88 treatment. EAE was induced by using homologous homogenates extracted from the brain and spinal cord of SD rats [18]. Homogenates containing 1 g of tissue (including white matter from cerebellum and whole spinal cord) and 1 ml of phosphate buffered saline (PBS) were emulsified in 1 ml of complete Freund’s adjuvant (Sigma-Aldrich, St. Louis, MO, USA) with 5 mg/ml of *Mycobacterium tuberculosis* (H37RA). These rats were subcutaneously injected in the footpad with this emulsion at a dose of 150 μl/200 g and with an auxiliary injection of 0.2 ml of pertussis toxin. The rats were randomly divided into four groups (n = 16 in each group): (1) In the control group, the rats received only PBS. (2) In the EAE group, the rats received the emulsion and pertussis toxin for immunization, and the second immunization was administered 7 days after the first injection. (3) In the prevention group, the rats were injected daily with ImKTx88 subcutaneously (100 μg/kg in 1 ml PBS) from day 0 to day 23. (4) In the treatment group, the rats were administered ImKTx88 (100 μg/kg in 1 ml PBS) after the onset of disease. The clinical scores were recorded daily as follows [54]: 0 = no detectable changes in clinical signs; 1 = limp tail; 2 = hind limb paresis weakness; 3 = hind limb paralysis; 4 = fore limbs and hind limbs were all paralyzed; 5 = moribund or death. Special care was required for animals with severe EAE (score of 3 or more).

### Cell culture

Peripheral blood mononuclear cells were isolated from whole blood samples of the experimental rats by Percoll (Sigma-Aldrich, St Louis, MO, USA) gradient centrifugation at 400*g* for 25 min. The PBMCs were seeded in 96-well plates at 10^6^ cells/well and were cultured in 1640 RPMI medium containing 10% fetal bovine serum (FBS, Pan, Germany), 100 U/ml penicillin and 100 μg/ml streptomycin (Beyotime Biotechnology, China) at 37 °C. The cells were divided into five groups. In the stimulation group, the cells were stimulated with 100 μg/ml Concanavalin A (Con A, Sigma-Aldrich, St. Louis, MO, USA) for 24 h. In the treatment groups, the cells were incubated with different concentrations of ImKTx88 (100 nM, 1, and 10 μM) 60 min before Con A stimulation. The control cells were given PBS treatment without any stimulation. Those cells were cultured for 24 h before harvesting.

### Evans blue extravasation measurement

Blood–brain barrier leakage was assessed using Evans blue as previously described [55]. On day 21 post-immunization (the peak of the disease), six rats from each group were anesthetized. 2% Evans blue dye (Sigma-Aldrich, St. Louis, MO, USA) was slowly injected into the tail vein (4 ml/kg) and was allowed to circulate for 60 min. To flush excess dye, the rats were perfused with 150 ml of saline. To verify systemic dye distribution, the kidney tissue was extracted to confirm successful circulation. When the rats were sacrificed, the brains and the lumbar enlargement of the spinal cords were obtained and were immediately weighed. Evans blue extraction for all samples was processed in parallel. Tissues (n = 6) were immersed and homogenized in 5 ml of formamide and were maintained at room temperature for 48 h. Samples were centrifuged at 14,500*g* for 30 min, and the supernatant was removed for analysis. The absorbance of the supernatant at 630 nm was measured with a microplate reader (Bio-tek Elx800, USA), and the Evans blue dye content was quantified as μg/g brain tissue.

### Histopathology and immunohistochemistry

The rats were sacrificed on day 21 following EAE induction. Brains were carefully removed and were fixed in 4% paraformaldehyde. Brain tissues were paraffin-embedded, and samples that contained the cerebellum regions were sectioned (4 μm). Some sections were stained with hematoxylin–eosin for the inflammatory infiltration test, and other sections were stained with Luxol fast blue (Google-BIO, Wuhan, China) for the demyelination test. Histopathological analysis was carried out and scored in a blinded fashion as follows. The severity of inflammation was scored according to previously published modified criteria on HE stains [56]. For the demyelination evaluation, we used a semi-quantitative scoring system as described previously [57]. For each animal, three histological sections were analyzed, and their average scores were calculated.

Other sections for immunohistochemistry underwent deparaffination, followed by rehydration and blocking with 10% normal goat serum (NGS) in PBS. Sections were incubated with antibodies for ICAM-1 (1:40, R&D, Boston, MA, USA) and VCAM-1 (1:50, Santa Cruz Biotechnology, CA, USA) overnight at 4°C. Then, the sections were incubated with secondary antibody and PAP complexes for 50 min at room temperature. The sections were visualized with DAB chromogenic reagent (ZSGB-BIO, Beijing, China) and counterstained with hematoxylin (Google-BIO, Wuhan, China). The slides were dehydrated and then coverslipped. Brain sections were visualized with a microscope (Nikon Eclipse TI-SR, Japan). The number of positive cells was calculated in a restricted area within the cerebellum and then analyzed using Image-Pro Plus 6.0 software.

### Immunofluorescent staining

Paraffin-embedded cerebellum tissue from each group was cut into 4 μm thick sections. Immunofluorescent staining was performed on these slices, which were incubated with anti-rat antibodies for claudin-5 (1:200, Invitrogen, Carlsbad, CA, USA) overnight at 4 °C. Sections were then washed and incubated with FITC-conjugated secondary antibodies in blocking solution for 1 h at room temperature and were mounted with DAPI Fluromount (Southern Biotechnology, Birmingham, AL, USA) and immediately observed with an inverted Zeiss Axioplan epifluorescence microscope (Germany). For quantification, images were imported into Image-Pro Plus 6.0 software.

### RNA isolation and quantitative real-time PCR (qRT-PCR)

Total RNA was isolated from the rat cerebellum using Trizol reagent (Invitrogen, Carlsbad, CA, USA). RNA was reverse-transcribed into cDNA using 2 μg of total RNA with a reverse transcription kit (Thermo Scientific, Waltham, MA, USA) with 20 μl of the reaction system. qRT-PCR was performed with SYBR Green Real-Time PCR Master Mix (Toyobo, Osaka, Japan) according to the manufacturer’s instructions. The qRT-PCR conditions for measuring mRNA levels in cerebellum tissues used in our laboratory have been reported previously [58]. The primers were as follows: occludin

F: CGGTACAGCAGCAACGATAA,

R: CTGTCGTGTAGTCGGTTTCATAG;

ZO-1

F: TTGCCACACTGTGACCCTAA,

R: GTTCACACTGCTTAGTCCAGC;

claudin-5

F: CGAGGCAAGTTAGGTTGGG,

R: GGTCGGTCAAGTCCTCACAA;

Ang-1

F: GCTGAACGGTTACACAGAGAG,

R: ACGCTCTCCCCGTTAAAGAAA;

Tie-2

F: AGCAGGAGCAGATAAGCGTT,

R: CACTTGGTATCAGCAGGGCT;

IL-17

F: ACGCCGAGGCCAATAACTTT,

R: AGAGTCCAGGGTGAAGTGGA;

GAPDH

F: CAAGGATACTGAGAGCAAGAGAGA,

R: TCCTGTTGTTATGGGGTCTGG.

Quantitative PCR was performed with the Bio-Rad CFX96 Real-Time PCR System (Life Technologies, Norwalk, CT, USA). Expression levels were normalized to GAPDH. The results were analyzed with the Bio-Rad CFX96 software and the 2^−∆∆ct^ method.

### Western blotting analysis

The freshly isolated cerebellum tissues were collected and homogenized. Protein content was determined with a BCA Protein Quantitation Kit (Thermo Fisher Scientific, Waltham, MA, USA). A total of 10–30 μg protein was loaded in each lane and was separated by 10% sodium dodecyl sulfate–polyacrylamide gel electrophoresis (SDS-PAGE). After separation, the proteins were transferred to polyvinylidene difluoride (PVDF) membranes (Millipore, Hertfordshire, UK). The membranes were blocked with 5% bovine serum albumin. Blots were incubated overnight with the primary antibodies as follows: occludin (1:1000, Abcam, Cambridge, MA, USA); ZO-1 (1:500, Invitrogen, Carlsbad, CA, USA); claudin-5 (1:500, Abclonal, Woburn, MA, USA); ICAM-1(1:1000, Abclonal, Woburn, MA, USA); VCAM-1 (1:1000, Abclonal, Woburn, MA, USA); Ang-1 (1:500, Abclonal, Woburn, MA, USA); Tie-2 (1:500, Abclonal, Woburn, MA, USA); GAPDH (1:5000, Abclonal, Woburn, MA, USA). Following 45–60 min incubation with horseradish peroxidase-conjugated rabbit or mouse secondary antibody (1:10,000, BioPM, Wuhan, China), blots was visualized using the enhanced chemiluminescence (ECL) detection system (Thermo Scientific, Waltham, MA, USA). Band densities were measured by densitometry and were quantified with Image J software (version 1.46r, Bethesda, MD, USA). GAPDH was used as a control.

### Enzyme-linked immunosorbent assay (ELISA)

Supernatants from PBMC cultures were collected 24 h after antigen stimulation. IL-17 levels in the supernatants were measured with rat Quantikine kits (R&D Systems, Boston, MA, USA) according to the manufacturer’s instructions. For the animal experiment, the IL-17 levels of cerebellar homogenates from experimental animals (at the peak stage of EAE) were also assessed with rat Quantikine kits (R&D Systems, Boston, MA, USA). Samples and standards were tested in duplicate, and every sample data point was compared to a standard curve from these kits to determine IL-17 levels.

### Statistical analysis

All data are presented as the mean ± SEM. Statistical significance was analyzed using Graph Pad PRISM 5.0 software (Graph Pad Software, Inc. La Jolla, CA, USA). For non-parametric data with multiple groups, the clinical scores and histopathologic scores were assessed using one-way non-parametric ANOVA (Kruskal–Wallis test) for independent data. For the other data, differences between multiple groups were analyzed by one-way ANOVA. Differences were considered significant if *P* was <0.05.

## Additional file

**Additional file 1: Tables S1.** ImKTx88 reduces clinical signs in EAE rats.

## Abbreviations

BBB: blood–brain barrier
MS: multiple sclerosis
EAE: experimental autoimmune encephalomyelitis
TJ: tight junction
ZO-1: zonula occludens protein 1
ICAM-1: intercellular adhesion molecule 1
VCAM-1: vascular cell adhesion molecule 1
Ang-1: angiopoietin-1
Tie-2: endothelial-specific receptor tyrosine kinase 2
HE: hematoxylin-eosin
LFB: Luxol fast blue
Con A: Concanavalin A
PBMCs: peripheral blood mononuclear cells
IOD: integrated optical density
