# Supplementary material for: Kv1.3 channel blocker (ImKTx88) maintains blood–brain barrier in experimental autoimmune encephalomyelitis
Source: Cell Biosci. 2017 Jun 7;7:31. doi: 10.1186/s13578-017-0158-2 (PMC5463463; doi:10.1186/s13578-017-0158-2)
Supplement: Supplementary file 1 — Additional file 1: Tables S1. ImKTx88 reduces clinical signs in EAE rats. [file 13578_2017_158_MOESM1_ESM.docx]

**Table S1 ImKTx88 reduces clinical signs in EAE rats**

| **Days** | **EAE** | **Groups**  **Prevention** | **Treatment** |
| --- | --- | --- | --- |
| 12 | 0.03 ± 0.13 | 0.00 ± 0.00 | 0.00 ± 0.00 |
| 13 | 0.06 ± 0.25 | 0.00 ± 0.00 | 0.00 ± 0.00 |
| 14 | 0.19 ± 0.40 | 0.00 ± 0.00 | 0.06 ± 0.25 |
| 15 | 0.56 ± 0.89 | 0.00 ± 0.00 | 0.13 ± 0.50 |
| 16 | 1.38 ± 1.71 | 0.00 ± 0.00 | 0.28 ± 0.68 |
| 17 | 1.63 ± 1.93 | 0.13 ± 0.50 | 0.31 ± 0.70 |
| 18 | 2.00 ± 2.00 | 0.44 ± 0.96 | 0.63 ± 1.02 |
| 19 | 2.56 ± 1.82 | 0.75 ± 1.18 | 0.88 ± 1.45 |
| 20 | 2.94 ± 1.88 | 0.94 ± 1.18 | 1.13 ± 1.31 |
| 21 | 3.06 ± 1.77 | 0.75 ± 1.06 | 1.25 ± 1.34 |
| 22 | 3.00 ± 1.79 | 0.50 ± 0.82 | 1.13 ± 1.41 |
| 23 | 3.00 ± 1.79 | 0.44 ± 0.81 | 0.75 ± 1.06 |

**Table S1** ImKTx88 reduces clinical signs in EAE rats. Data represent the mean ± SEM (n=16).
